# Pyruvate Carboxylase Attenuates Myocardial Ischemia–Reperfusion Injury in Heart Transplantation via Wnt/β-Catenin-Mediated Glutamine Metabolism

**DOI:** 10.3390/biomedicines12081826

**Published:** 2024-08-12

**Authors:** Zihao Wang, Hongwen Lan, Yixuan Wang, Qiang Zheng, Chenghao Li, Kan Wang, Tixiusi Xiong, Qingping Wu, Nianguo Dong

**Affiliations:** 1 Department of Cardiovascular Surgery, Union Hospital, Tongji Medical College, Huazhong University of Science and Technology, Wuhan 430014, China; whuhwangzihao@163.com (Z.W.);; 2 Department of Thoracic Surgery, Wuhan No. 1 Hospital, Tongji Medical College, Huazhong University of Science and Technology, Wuhan 430014, China; 3 Department of Anesthesiology, Union Hospital, Tongji Medical College, Huazhong University of Science and Technology, Wuhan 430014, China

**Keywords:** pyruvate carboxylase, ischemia–reperfusion injury, Wnt/β-catenin pathway, glutamine metabolism, heart transplantation

## Abstract

The ischemia–reperfusion process of a donor heart during heart transplantation leads to severe mitochondrial dysfunction, which may be the main cause of donor heart dysfunction after heart transplantation. Pyruvate carboxylase (PC), an enzyme found in mitochondria, is said to play a role in the control of oxidative stress and the function of mitochondria. This research examined the function of PC and discovered the signaling pathways controlled by PC in myocardial IRI. We induced IRI using a murine heterotopic heart transplantation model in vivo and a hypoxia–reoxygenation cell model in vitro and evaluated inflammatory responses, oxidative stress levels, mitochondrial function, and cardiomyocyte apoptosis. In both in vivo and in vitro settings, we observed a significant decrease in PC expression during myocardial IRI. PC knockdown aggravated IRI by increasing MDA content, LDH activity, TUNEL-positive cells, serum cTnI level, Bax protein expression, and the level of inflammatory cytokines and decreasing SOD activity, GPX activity, and Bcl-2 protein expression. PC overexpression yielded the opposite findings. Additional research indicated that reducing PC levels could block the Wnt/β-catenin pathway and glutamine metabolism by hindering the movement of β-catenin to the nucleus and reducing the activity of complex I and complex II, as well as ATP levels, while elevating the ratios of NADP+/NADPH and GSSG/GSH. Overall, the findings indicated that PC therapy can shield the heart from IRI during heart transplantation by regulating glutamine metabolism through the Wnt/β-catenin pathway.

## 1. Introduction

Heart transplantation is still the primary form of curative therapy for individuals suffering from advanced heart failure. However, despite advancements made in operative techniques and postoperative management in recent years, ischemia–reperfusion injury (IRI) remains a major challenge in organ transplantation [1,2]. Severe myocardial IRI, which typically occurs in hearts subjected to prolonged cold ischemia time, has been associated with primary graft dysfunction and mortality following heart transplantation [3,4,5,6]. Therefore, new strategies are needed to alleviate myocardial IRI in donor hearts during heart transplantation.

Pyruvate carboxylase (PC) is a mitochondrial enzyme that requires biotin and is found in many tissues. It plays a role in converting pyruvate to oxaloacetate through carboxylation. The carboxylation process is a significant anaplerotic route through which alanine and lactate restore tricarboxylic acid (TCA) cycle compounds. Significant changes in the protein expression of PC have been documented in metabolic syndrome and cancer progression [7,8,9]. The potent antioxidant capacity of PC has attracted significant interest and concern in recent years [10,11]. Alleviating oxidative stress is widely thought to be effective against myocardial IRI. Nevertheless, there is insufficient research on the impact of PC in heart transplantation during IRI.

Glutamine, a plentiful free amino acid in the body, makes up about 40% of the free amino acid pool in cardiac muscle [12]. Abnormal glutamine metabolism is present in failing myocardium, suggesting a pivotal role of glutamine metabolism in cardiac homeostasis [13]. Growing evidence suggests that enhanced glutamine metabolism can attenuate myocardial IRI [14,15,16]. Furthermore, Fu et al. revealed that PC is necessary and sufficient for glucose metabolism to produce glutathione and respond to oxidative stress [17]. It is still uncertain whether this pathway is essential for the cardioprotective benefits of PC in myocardial IRI.

The current research examined how PC impacts myocardial IRI in heart transplants and explored how glutamine metabolism plays a role in the cardioprotective benefits of PC.

## 2. Materials and Methods

### 2.1. Animals

Approval number 3087 was granted by the Ethics Committee on Animal Care of Union Hospital, Tongji Medical College, Huazhong University of Science and Technology, for all animal experimental procedures. Male C57BL/6 mice (8–12 weeks old, weighing 22–28 g) were purchased from Huafukang Experimental Animal Center, Beijing, China. The animals were kept in a sterile environment with a 12 h light/dark cycle at a temperature of 23 ± 1 °C and were given unrestricted access to a standard pellet diet throughout the study.

Each mouse received an injection via the tail vein of 1 × 10^11^ vector genomes of adeno-associated virus (AAV) serotype 9 to investigate the effects of PC overexpression or knockdown with a single dose. The AAV contained genes such as AAV-PC, AAV-sh-PC, AAV-GFP, and AAV-sh-Con. Mice underwent further experimentation after four weeks of AAV injection. The study utilized AAVs that were created and acquired from Dianjun Biotechnology in Shanghai, China.

### 2.2. Reagents

Kits for assessing levels of malondialdehyde (MDA), superoxide dismutase (SOD) activity, glutathione peroxidase (GPX) activity, caspase-3 activity, ATP levels, reactive oxygen species (ROS) production, and bicinchoninic acid (BCA) protein assay were acquired from Beyotime Biotechnology in Shanghai, China. Commercially available kits from Nanjing Jiancheng Bioengineering Institute in China were used to measure interleukin-1β (IL-1β), interleukin-6 (IL-6), monocyte chemoattractant protein-1 (MCP-1), tumor necrosis factor (TNF-α), and lactate dehydrogenase (LDH). Commercially available kits (Sigma, St. Louis, MO, USA) were used to assay NADP+/NADPH ratio and GSSG/GSH ratio. The activity of respiratory chain complexes I and II was measured using kits from Cayman in Ann Arbor, MI, USA. MSAB was acquired from MCE in Shanghai, China. The radioimmunoprecipitation assay (RIPA) lysis buffer, containing 50 mM Tris-HCl, 150 mM sodium chloride, 1% Triton X-100, 1% sodium deoxycholate, and 0.1% sodium dodecyl sulfate, along with protease inhibitor, was purchased from Servicebio in Wuhan, China. Antibodies against Bax (32503), GAPDH (ab8245), PC (ab123451), c-Myc (EPR17924), Cyclin D1 (ab16663), histone H3 (ab1791), and glutamine synthase (GS, ab313449) were obtained from Abcam (Cambridge, UK). Proteintech (Wuhan, China) provided antibodies for Bcl-2 (26593-1-Ap) and β-catenin (17565-1-Ap), with β-tubulin (2146) antibodies acquired from Cell Signaling Technology (Danvers, MA, USA).

### 2.3. Retrieval and Preservation of Donor Hearts

Donor hearts were obtained in the same manner as detailed in a previous study [18]. Following the preparation of the perfusate, the mice were anesthetized using isoflurane through inhalation, with 5% for induction and 2.5% for maintenance of anesthesia. Heparinization was carried out systemically at a dose of 400 IU/kg through the inferior vena cava. The University of Wisconsin (UW) solution was used to perfuse the heart, causing cardiac arrest. After that, the heart was harvested using conventional techniques and stored in UW solution at 4 °C. Donor hearts from mice in the IRI group were subjected to heart transplantation after 24 h of cold preservation, while donor hearts from mice in the control group were transplanted instantly without cold preservation.

### 2.4. Heterotopic Heart Transplantation

A previous study detailed the process of conducting syngeneic murine heterotopic heart transplantation [19]. In short, mice were given anesthesia through inhalation of isoflurane (5% for induction; 2.5% for maintenance). Heart grafts were harvested from donor mice and transplanted into the abdominal cavity of recipient mice via the end-to-side anastomosis of the aorta and pulmonary artery of the graft to the recipient aorta and vena cava, respectively. The duration of obtaining the donor heart was limited to less than 10 min to mitigate the effects caused by significant warm ischemic injury. The process of suturing the donor heart to the recipient during transplantation was limited to less than 40 min.

### 2.5. Neonatal Rat Cardiomyocyte Isolation and Culture

NRCMs were obtained from newborn Sprague–Dawley rats within the first three days of life. The ventricle tissue was quickly collected, placed in ice-cold Hanks solution to eliminate blood, chopped into small pieces, and then exposed to a digestion solution containing 0.1% trypsin and 1 mg/mL collagenase type II at 4 °C for the entire night. The following day, the processed tissue was moved to a plate and then gathered into a cone-shaped tube. The heart tissue was treated with the digestion solution and then incubated at 37 °C for 15 min while gently shaking. The liquid above the cells was moved to a new mixture of DMEM with 20% FBS, while the heart tissue that was not broken down was mixed back into the digestion solution. The process of digestion was performed five times, resulting in the collection of the supernatant-containing cells. Following purification through Percoll density-gradient centrifugation, NRCMs were placed in a humid incubator with 5% CO_2_ at a temperature of 37 °C. The culture medium contained 10% FBS and 1% penicillin/streptomycin.

### 2.6. In Vitro Hypoxia–Reoxygenation Model

An H/R model was created based on a prior study [20]. The UW solution was used to replace the cell culture medium, and then, the cells were transferred to a hypoxia incubator bag at 4 °C with 0.1% O_2_. Following 24 h of low oxygen levels, the cells were placed back into standard culture conditions (95% oxygen; 5% carbon dioxide), and the medium was switched to full DMEM (including 10% fetal bovine serum and 1% penicillin/streptomycin) for an additional 6 h to simulate the reperfusion stage in a laboratory setting. Cells grown under the standard cell culture conditions were utilized as the normoxia control.

### 2.7. Adenovirus Construction and Transduction

Dianjun Biotechnology (Shanghai, China) produced and increased the number of recombinant adenoviruses containing rat PC (Ad-PC) and recombinant adenoviruses carrying an improved green fluorescent protein (Ad-GFP). NRCMs were cultured until reaching 60–70% confluency, then infected with adenoviruses at an MOI of 50 for 24 h. The transfection process was stopped for 24 h before proceeding with additional experiments.

### 2.8. RNA Interference In Vitro

PC siRNA (Ruibo Biotechnology, Guangzhou, China) or β-catenin siRNA (Ruibo Biotechnology, Guangzhou, China) was transfected into NRCMs for RNA interference. The siRNA-targeting sequences of PC and β-catenin were GAATACTCGTCTCTTTCTT and GCACCAUGCAGAAUACAAA, respectively. Transfection was initiated using LipofectamineTM 3000 Transfection Reagent (Thermo Fisher Scientific, Waltham, MA, USA) in Opti-MEM for 12 h, followed by replacement with complete DMEM for an additional 12 h in preparation for subsequent experiments.

### 2.9. Luciferase Reporter Assay

In order to identify how β-catenin affects the transcriptional control of GS, the GS promoter reporter plasmid was introduced into HEK-293 cells along with either the β-catenin-overexpression plasmid or a control vector. The firefly luciferase emitted a signal detected at 560 nm, while the renilla luciferase emitted a signal detected at 465 nm. Relative luciferase activity was determined by calculating the ratio of the firefly luciferase signal to the renilla luciferase signal.

### 2.10. Terminal Deoxynucleotidyl Transferase Dutp Nick-End Labeling Assay

Apoptosis in the myocardium of the left ventricle was assessed using the Roche (Basel, Switzerland) TUNEL assay following the provided guidelines.

### 2.11. Mitochondrial Oxidative Stress Evaluation, Inflammatory Cytokines Assessment, LDH and cTnI Detection

Commercially available kits were used to measure indicators of oxidative stress in mitochondria, such as MDA content, SOD and GPX activities, and LDH activity, in accordance with the manufacturer’s instructions. Following the heart transplant, a plasma specimen was obtained for analysis of cTnI and inflammatory cytokines such as IL-1β, IL-6, MCP-1, and TNF-α utilizing kits that are readily available for purchase. The levels of ATP, ratios of NADP+/NADPH and GSSG/GSH, and the activities of respiratory chain complexes I and II were assessed following the guidelines provided by the manufacturer.

### 2.12. Intracellular Reactive Oxygen Species Detection

Frozen cardiac tissue sections were placed on glass slides and treated with dihydroethidium (DHE) to observe the production of reactive oxygen species (ROS) in the heart during ischemia–reperfusion injury (IRI). Microscopy was used to determine the proportion of areas stained with DHE on cryosections after staining with DHE for 30 min at 37 °C.

### 2.13. Real-Time Quantitative PCR

Total RNA was extracted from NRCMs using Trizol reagent (Thermo Fisher Scientific) and subjected to reverse transcription using the RT Reagent Kit (Vazyme Biotech, Nanjing, China) to obtain cDNA. Real-time PCR assays were performed using SYBR Green (Vazyme Biotech, Nanjing, China) to measure the levels of GS mRNA on Applied Biosystems equipment from Thermo Fisher Scientific in the USA. Normalization of mRNA expression to GAPDH was performed using the 2^−∆∆ct^ method. The primers used in this study were GS-F 5′-TGAATGTGACGCCAGGTGAG-3′ and GS-R 5′-GTCCAGAGTACGGGTCTTGC-3′, GAPDH-F 5′-GCATCTTCTTGTGCAGTGCC-3′ and GAPDH-R 5′-GATGGTGATGGGTTTCCCGT-3′. All the primers were designed by AuGCT DNA-SYN Biotechnology Co, Ltd. (Wuhan, China).

### 2.14. Western Blot Analysis

The liquid nitrogen was used to preserve a sample of the left ventricle for Western blot analysis. The heart tissue was quickly crushed and broken down in RIPA lysis buffer containing a protease inhibitor mix. The BCA protein assay kit was used to measure protein levels. Proteins were isolated using sodium dodecyl sulfate–polyacrylamide gel electrophoresis and then transferred to polyvinylidene difluoride membranes. The blots were subsequently treated with primary antibodies targeting Bax, Bcl-2, PC, β-catenin, c-Myc, Cyclin D1, histone H3, GS, β-tubulin, and GAPDH. The next day, the filters were rinsed with tris-buffered saline tween (10 mM Tris-base, 100 mM sodium chloride, and 0.1% tween-20, pH 7.50) and then exposed to suitable secondary antibodies for 1 h at ambient temperature. Quantification of the protein bands was performed with the assistance of the Image Lab 6.0.1 software system.

### 2.15. Statistical Analysis

Data were expressed as means ± standard deviations of the mean (SD) values. A Shapiro–Wilk test was used to assess the normality distribution of variables. A two-tailed parametric t-test was used to evaluate variations in the average values of the two groups, assuming a normal distribution of values. Non-normally distributed variables between two groups were compared using a non-parametric Mann–Whitney U test. A substantial distinction was determined when *p* was less than 0.05. SPSS 26.0 (IBM Corporation, Armonk, NY, USA) was utilized for the analyses.

## 3. Results

### 3.1. PC Is Significantly Downregulated during Myocardial IRI

**Figure 1:**
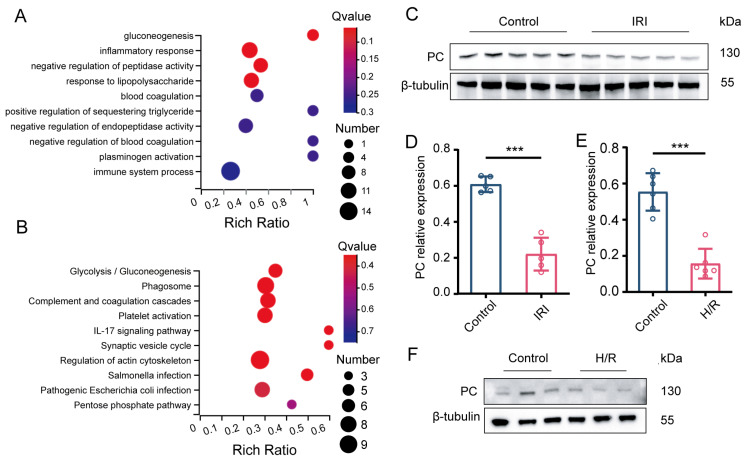
PC is significantly downregulated during myocardial IRI. (**A**) Enrichment analysis of top 10 GO terms for both the control and IRI groups. (**B**) Analysis of top 10 KEGG pathways in the control group and the IRI group. (**C**,**D**) Protein expression levels of PC in hearts subjected to IRI (*n* = 5). (**E**,**F**) Protein expression levels of PC in NRCMs subjected to H/R (*n* = 6). Data are shown as the means ± SD. *** *p* < 0.001 for indicated comparisons.

To identify the molecular changes following myocardial IRI, we performed a proteomics analysis on three pairs of left ventricular cardiac tissue samples from mice that underwent heart transplantation, with or without 24 h cold preservation. Pathway analysis identified gluconeogenesis as the most enriched pathway among the altered pathways (Figure 1A,B). In order to clarify the relationship between PC and myocardial ischemia–reperfusion injury, we first examined the protein expression levels of PC in an in vivo model before and after ischemia–reperfusion. Cardiac PC protein expression was found to be markedly reduced following IRI as shown by Western blot analysis (*p* < 0.001; Figure 1C,D). Furthermore, we assessed the protein expression of PC during H/R in our NRCMs model. PC protein expression was also significantly reduced in NRCMs subjected to H/R (*p* < 0.001; Figure 1E,F). Collectively, these findings indicate that PC could have a crucial impact on myocardial IRI in heart transplant procedures.

### 3.2. PC Knockdown Aggravates Myocardial IRI in Heart Transplantation

**Figure 2:**
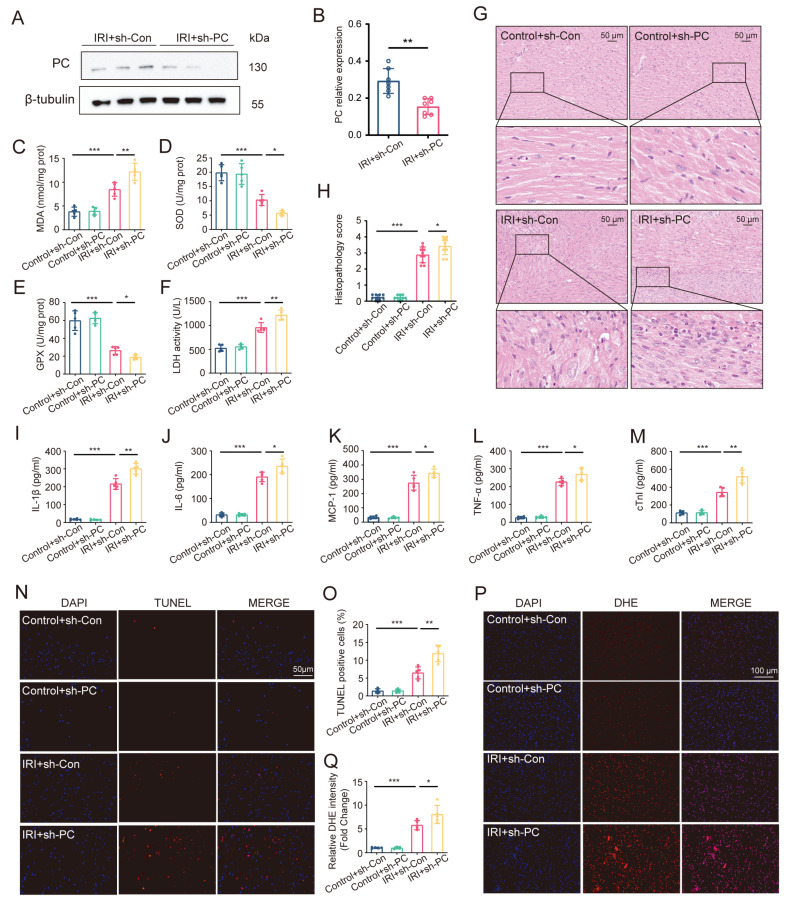
PC knockdown aggravates myocardial IRI in heart transplantation. (**A**,**B**) Protein levels of PC in cardiac tissue treated with AAV-sh-Con/AAV-sh-PC after IRI during heart transplantation (*n* = 6). The levels of (**C**) MDA content, (**D**) SOD activity, (**E**) GPX activity, and (**F**) LDH activity were detected in the hearts subjected to myocardial IRI (*n* = 5). (**G**,**H**) Heart inflammation was evaluated by H&E staining at 20× magnification with a scale bar of 50 μm. (**I**–**L**) Serum levels of inflammatory cytokines such as IL-1β, IL-6, MCP-1, and TNF-α were measured in the heart after myocardial IRI (*n* = 5). (**M**) The levels of serum cTnI were evaluated in hearts following IRI (*n* = 5). (**N**,**O**) Fluorescent pictures from the TUNEL test were taken for every group. Apoptotic heart muscle cells were treated with TUNEL staining (red), while the nuclei of all heart muscle cells were stained with DAPI (blue). The apoptotic index was calculated by determining the percentage of cells undergoing apoptosis compared to the total number of cells. (**P**,**Q**) Fluorescent pictures showing the generation of reactive oxygen species in every category (magnified by 200 times; scale bar of 100 μm). ROS underwent DHE staining, appearing red, while DAPI was used to stain the nuclei of all cardiomyocytes, appearing blue. Data are shown as the means ± SD. * *p* < 0.05, ** *p* < 0.01, *** *p* < 0.001 for indicated comparisons.

In order to verify the involvement of PC in myocardial IRI, we employed AAV-mediated methods to reduce its function, leading to successful PC knockdown (*p* < 0.01; Figure 2A,B). Following heart transplantation, there was a notable rise in MDA levels and LDH content, along with a decline in SOD and GPX functions in the cardiac tissue, suggesting the onset of oxidative stress due to IRI (*p* < 0.001; Figure 2C–F). Reducing PC expression led to higher MDA and LDH levels while also lowering antioxidative capacity through decreased SOD and GPX activities. Similarly, the PC knockdown group showed a significant exacerbation in myocardial inflammation (Figure 2G,H). Furthermore, reducing PC expression increased the concentrations of pro-inflammatory cytokines like IL-1β, IL-6, MCP-1, and TNF-α (Figure 2I–L). Moreover, the myocardial injury was aggravated by PC knockdown, as evidenced by the increase in TUNEL-positive cells (*p* < 0.01; Figure 2N,O). This observation was confirmed by the increased serum cTnI level (*p* < 0.01; Figure 2M). We next determined the role of PC during myocardial IRI by detecting ROS levels and conducting H&E staining. The IRI + sh-PC group exhibited a notable rise in ROS release levels compared to the IRI + sh-Con group (*p* < 0.05; Figure 2P,Q). These results suggest that PC knockdown could aggravate myocardial IRI during heart transplantation.

### 3.3. PC Knockdown Aggravates H/R Injury In Vitro

**Figure 3:**
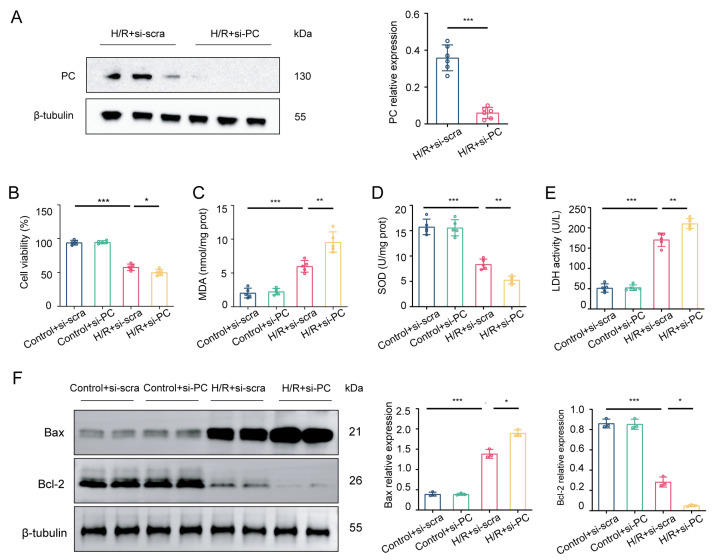
PC knockdown aggravates H/R injury in vitro. (**A**) Protein levels of PC expression in NRCMs transfected with NC siRNA or PC siRNA were measured after H/R (*n* = 6). (**B**) PC siRNA’s impact on NRCMs cell viability, (**C**) MDA content, (**D**) SOD activity, and (**E**) LDH activity was measured using kits (*n* = 5). (**F**) Western blotting was used to detect the protein levels of Bax and Bcl-2 (*n* = 3). Data are shown as the means ± SD. * *p* < 0.05, ** *p* < 0.01, *** *p* < 0.001 for indicated comparisons.

We next confirmed the role of PC in H/R through PC knockdown in NRCMs (Figure 3A). PC knockdown reduced cell viability, increased MDA levels, and decreased SOD activity (Figure 3B–D). Additionally, the enhancement of LDH activity indicated severe cell damage during H/R under PC knockdown (*p* < 0.01; Figure 3E). Reduced PC levels worsened myocardial cell death, shown by higher Bax levels and lower Bcl-2 levels in cardiomyocytes (*p* < 0.05; Figure 3F). Overall, these results suggest that PC knockdown aggravates H/R injury in NRCMs.

### 3.4. Knocking Down PC Suppresses the Wnt/β-Catenin Pathway and Glutamine Metabolism

**Figure 4:**
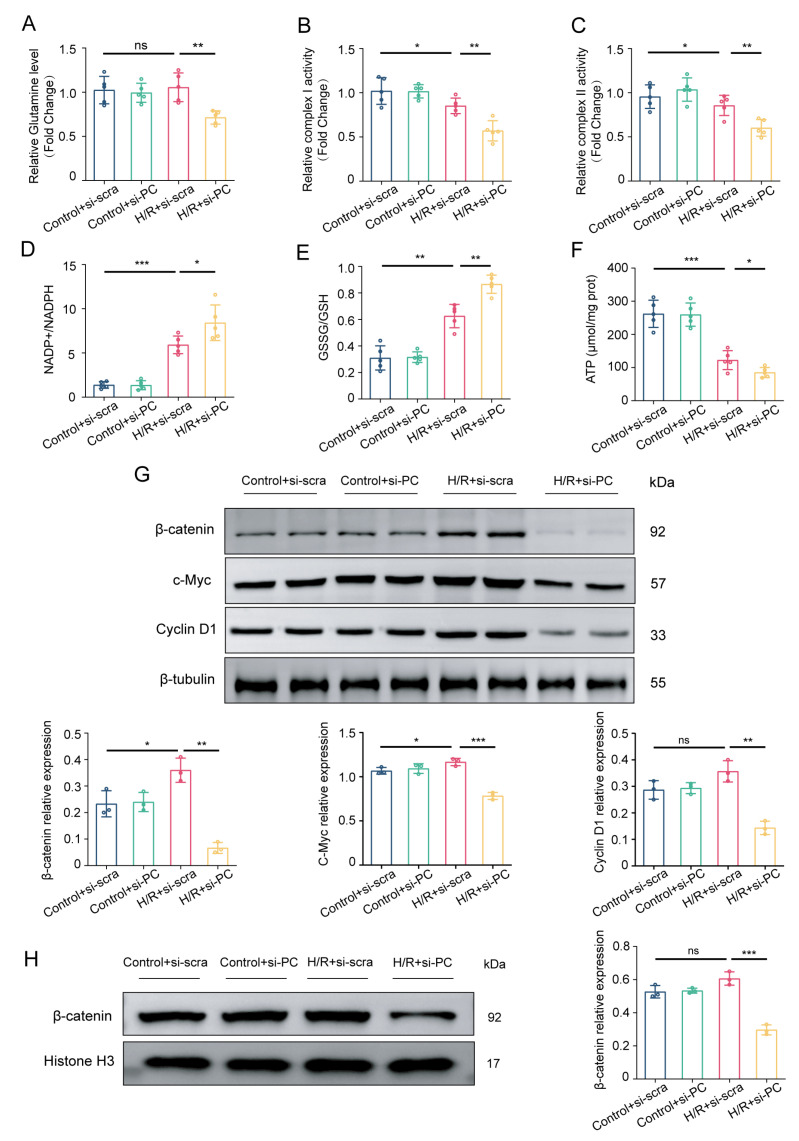
PC knockdown inhibits Wnt/β-catenin pathway and glutamine metabolism during H/R. (**A**) The glutamine levels were measured in NRCMs transfected with either scramble siRNA or PC siRNA after exposure to H/R, with a sample size of 5. (**B**,**C**) Complex I and complex II activities were measured in all groups. (**D**–**F**) The levels of NADP+/NADPH, GSSG/GSH, and ATP content in NRCMs following H/R were measured using kits (*n* = 5). (**G**) Western blotting was used to detect the protein levels of β-catenin, c-Myc, and Cyclin D1 (*n* = 3). (**H**) The protein expressions of β-catenin in nuclei (*n* = 3). Data are shown as the means ± SD. * *p* < 0.05, ** *p* < 0.01, *** *p* < 0.001 for indicated comparisons. n.s. signifies no significant difference for indicated comparisons.

PC is recognized to contribute to glutathione synthesis in response to oxidative stress damage [17]. Therefore, we determined alterations in glutamine metabolism. As shown in Figure 4A, PC knockdown decreased glutamine level. The downregulation of glutamine metabolism, an important pathway for replenishing cellular intermediates, has a detrimental effect on the function of mitochondrial oxidative phosphorylation. This impairment is evidenced by a substantial reduction in complex I activity, complex II activity, ATP content, and a significant increase in NADP+/NADPH and GSSG/GSH ratios (Figure 4B–F). Consistently, PC expression has been linked to the Wnt/β-catenin signaling pathway, with GS identified as a downstream target gene of this pathway, contributing to the regulation of glutamine metabolism [7,21]. We hypothesized that reducing PC levels would impact the Wnt/β-catenin signaling pathway in NRCMs exposed to H/R injury, aiming to determine whether PC has cardioprotective effects via the Wnt signaling pathway. Hence, the levels of proteins associated with the Wnt/β-catenin signaling pathway were measured. As shown in Figure 4G, the levels of β-catenin, c-Myc, and Cyclin D1 proteins in the H/R + si-PC group were lower compared to those in the H/R + si-scra group. After recognizing the necessity of the nuclear translocation of β-catenin for transcription, we proceeded to investigate its distribution within the nucleus. As shown in Figure 4H, the translocation of β-catenin into the nucleus was prevented by PC knockdown, suggesting inhibition of the Wnt/β-catenin pathway. Therefore, there is a strong possibility that PC provides protection for the heart by influencing glutamine metabolism through the Wnt/β-catenin pathway.

### 3.5. Wnt/β-Catenin Pathway Is Essential to Alleviate H/R Injury via PC Overexpression

**Figure 5:**
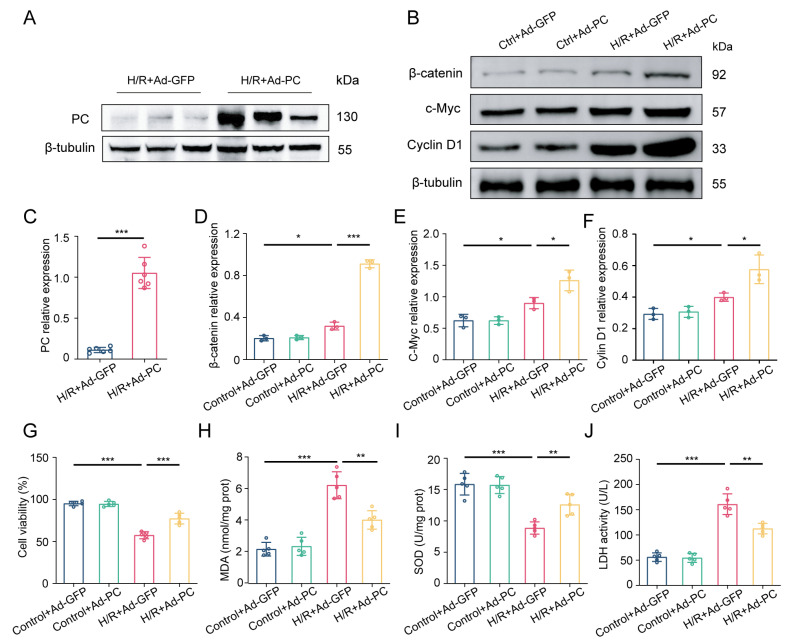
PC overexpression activates Wnt/β-catenin pathway during H/R. (**A**,**C**) Protein levels of PC expression in NRCMs transfected with Ad-GFP or Ad-PC after exposure to H/R were measured (*n* = 6). (**B**,**D**–**F**) Western blotting was used to detect the protein levels of β-catenin, c-Myc, and Cyclin D1 (*n* = 3). (**G**–**J**) Ad-PC impact on NRCMs cell viability, MDA content, SOD activity, and LDH activity was measured using kits (*n* = 5). Data are shown as the means ± SD. * *p* < 0.05, ** *p* < 0.01, *** *p* < 0.001 for indicated comparisons.

To further confirm the cardioprotective role of PC, we used AAV-mediated gain-of-function approaches (Figure 5A,C). Additionally, the overexpression of PC led to the notable stimulation of the Wnt/β-catenin pathway (Figure 5B,D–F). In comparison to the H/R + Ad-GFP group, the H/R + Ad-PC group showed decreased H/R injury through enhanced cell viability and mitochondrial ATP content, as well as increased SOD activity, along with decreased MDA and LDH activity (Figure 5G–J).

**Figure 6:**
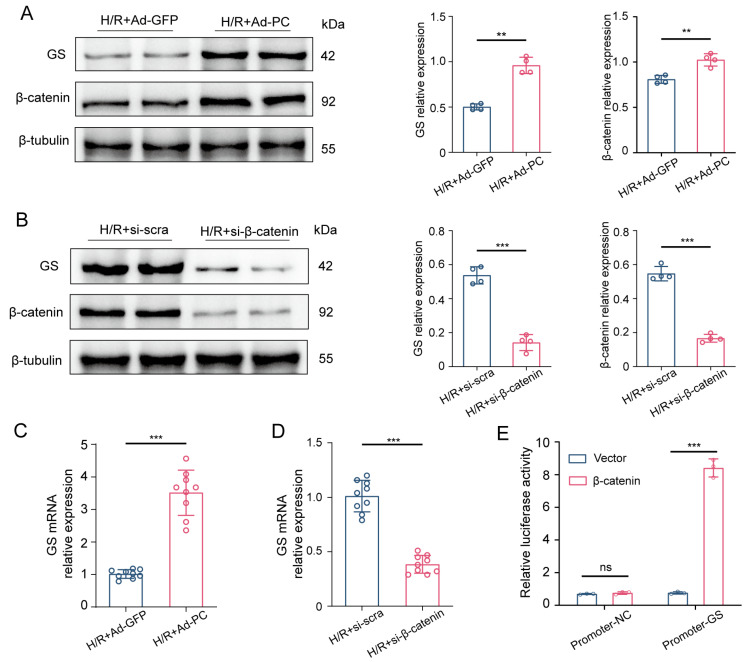
β-catenin upregulated GS expression by directly binding to its promoter. (**A**) Protein levels of GS and β-catenin were measured in NRCMs transfected with Ad-GFP or Ad-PC after H/R (*n* = 4). (**B**) Protein levels of GS and β-catenin were measured in NRCMs transfected with NC siRNA or β-catenin siRNA after H/R (*n* = 4). (**C**,**D**) GS mRNA expression (*n* = 9) in NRCMs. (**E**) A dual-luciferase assay was performed to assess β-catenin’s regulatory effect on the GS promoter. Data are shown as the means ± SD. ** *p* < 0.01, *** *p* < 0.001, and n.s. signifies no significant difference for indicated comparisons.

We investigated the impact of PC overexpression on glutamine metabolism via the Wnt/β-catenin pathway by analyzing GS expression in NRCMs transfected with Ad-PC after H/R. As shown in Figure 6A, PC overexpression not only increased β-catenin protein levels but also promoted GS protein expression. The decreased GS protein expression after β-catenin siRNA treatment indicated that β-catenin was likely to regulate GS expression (Figure 6B). The same result was further approved by the quantitative real-time PCR (Figure 6C,D). Dual-luciferase assay demonstrated that β-catenin could directly bind to the promoter of GS (Figure 6E). Overall, the findings indicated that β-catenin interacted with the GS promoter region to control glutamine metabolism.

**Figure 7:**
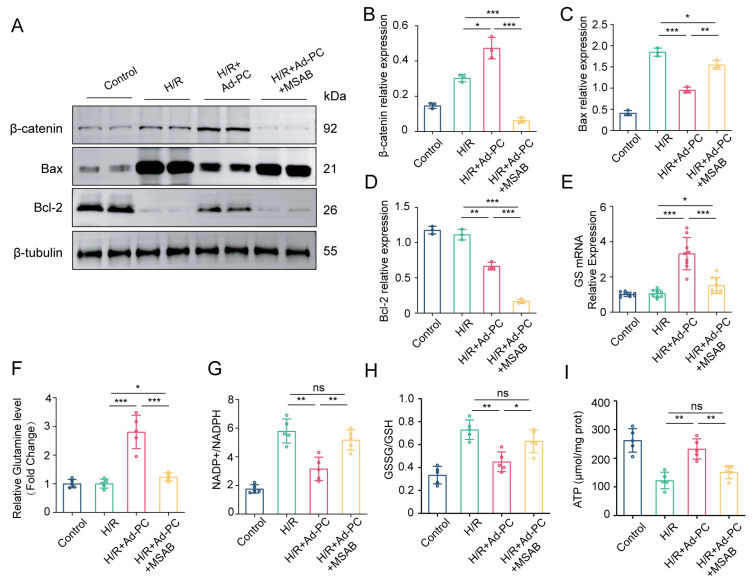
Effect of MSAB administration on H/R injury after PC treatment. (**A**–**D**) Western blotting was used to detect the protein levels of β-catenin, Bax, and Bcl-2 (*n* = 3). (**E**,**F**) GS mRNA expression (*n* = 9) and glutamine level (*n* = 5) in NRCMs were evaluated with MSAB administration after PC treatment. (**G**–**I**) The levels of NADP+/NADPH, GSSG/GSH, and ATP content of NRCMs after H/R were measured by kits (*n* = 5). Data are shown as the means ± SD. * *p* < 0.05, ** *p* < 0.01, *** *p* < 0.001, and n.s. signifies no significant difference for indicated comparisons.

Subsequently, we validated the critical function of the Wnt/β-catenin signaling pathway in H/R injury after overexpressing PC. As shown in Figure 7A–D, the administration of MSAB, a selective inhibitor of the Wnt/β-catenin pathway, for 12 h before H/R induction at a concentration of 5 μM, reversed the protective effects of PC on cardiomyocytes by increasing the expression of the pro-apoptotic factor Bax and decreasing the expression of the anti-apoptotic factor Bcl-2. Furthermore, MSAB administration suppressed glutamine metabolism and impaired mitochondrial function, as reflected in the significant decrease in GS mRNA expression, glutamine level, and ATP content, and a significant increase in NADP+/NADPH and GSSG/GSH ratios (*p* < 0.01; Figure 7E–I). The findings indicate that the Wnt/β-catenin pathway is essential for PC to reduce the H/R damage.

### 3.6. PC Alleviates Myocardial IRI during Heart Transplantation

**Figure 8:**
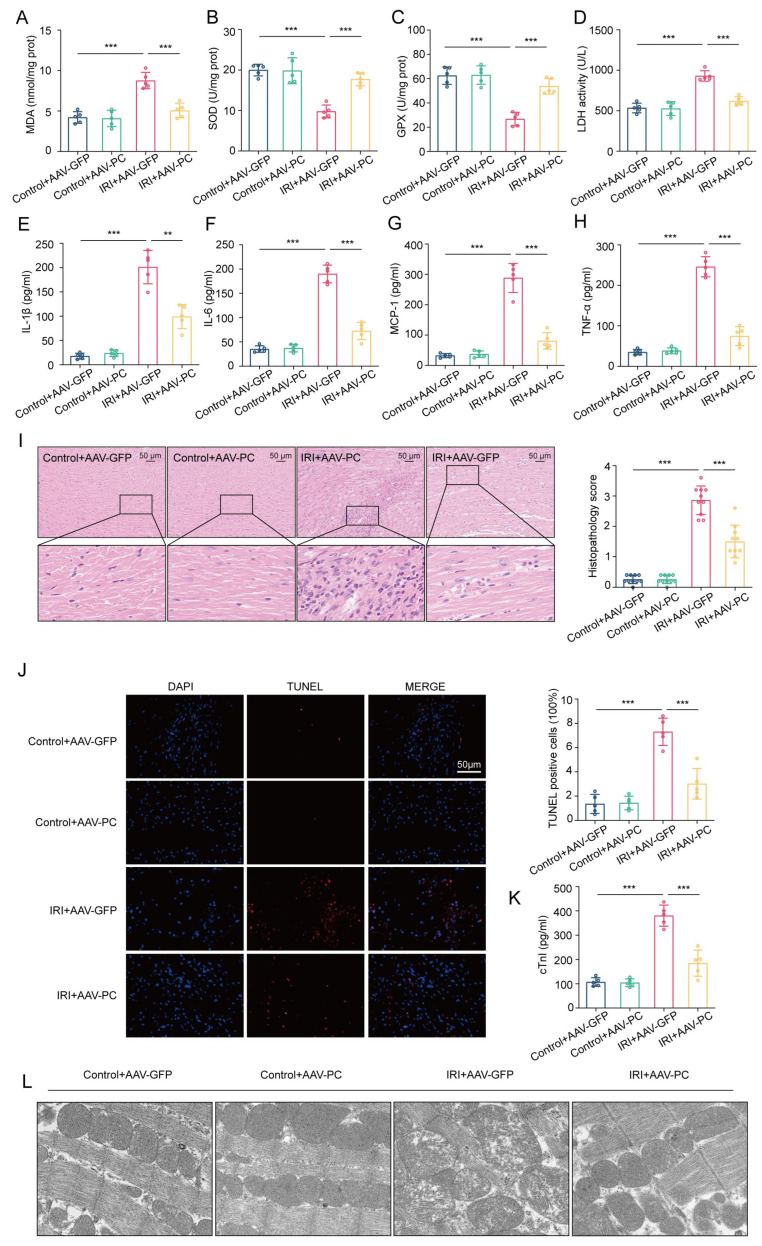
PC overexpression alleviates myocardial IRI in heart transplantation. (**A**–**D**) The levels of MDA content, SOD activity, GPX activity, and LDH activity were detected in the hearts treated with AAV-GFP/AAV-PC after IRI during heart transplantation (*n* = 5). (**E**–**H**) Serum levels of inflammatory cytokines such as IL-1β, IL-6, MCP-1, and TNF-α were measured in the hearts after myocardial IRI (*n* = 5). (**I**) Inflammation in the cardiac tissue was evaluated using H&E staining at a magnification of 20× with a scale bar of 50 μm. (**J**) Fluorescent pictures from the TUNEL test were taken for every group. Apoptotic heart muscle cells were treated with TUNEL staining (red), while the nuclei of all heart muscle cells were stained with DAPI (blue). The apoptotic index was calculated by determining the percentage of cells undergoing apoptosis compared to the total number of cells. (**K**) Serum cTnI levels were evaluated after IRI (*n* = 5). (**L**) Representative transmission electron microscopy images in each group. Data are shown as the means ± SD. ** *p* < 0.01, *** *p* < 0.001.

We continued our investigation into the cardioprotective effects of PC in vivo. As shown in Figure 8A–D, PC overexpression in the IRI + AAV-PC group significantly reduced MDA content and LDH levels while increasing SOD and GPX activities compared to the IRI + AAV-GFP group (*p* < 0.001). Furthermore, PC therapy led to a reduction in the levels of inflammatory cytokines including IL-1β, IL-6, MCP-1, and TNF-α (Figure 8E–H). The anti-inflammatory effect of PC was further confirmed by a decrease in inflammatory infiltrate (*p* < 0.001; Figure 8I). The reduction in apoptotic cardiomyocytes and serum cTnI levels indicated that PC treatment mitigated myocardial damage during IRI (*p* < 0.001; Figure 8J,K). Furthermore, the cardioprotective effects of PC were demonstrated at the microstructure level through transmission electron microscopy (Figure 8L). The structural abnormalities caused by IRI were attenuated after PC treatment. The findings indicate that PC plays a crucial role in reducing myocardial IRI in the mouse model of heterotopic heart transplantation.

## 4. Discussion

The current research delved into the protective impact of PC on heart transplants using AAV-induced methods to alter function. We also substantiated that PC was downregulated in the murine heterotopic heart transplantation model and NRCMs H/R model. Our findings showed that PC triggered the Wnt/β-catenin signaling pathway, leading to the control of glutamine metabolism, resulting in enhanced mitochondrial function, and helping alleviate myocardial IRI. This evidence outlined a novel molecular pathway involving Wnt/β-catenin that regulates glutamine metabolism, serving as a critical factor in preserving mitochondrial and cardiac function during heart transplantation. Promoting the activation of this pathway may protect the injured heart from dysfunction.

PC belongs to the biotin-dependent enzyme group, which is crucial in multiple cellular metabolic processes. The current understanding is that pyruvate carboxylase uses biotin as a coenzyme to facilitate the transformation of pyruvate into oxaloacetate. Oxaloacetate then enters the TCA cycle for the oxidative decomposition of glucose and fatty acids, leading to the accumulation of TCA cycle intermediates. Previous studies have corroborated that PC is involved in obesity, diabetes, viral infection, and cancer [22,23,24,25,26,27]. We showed that protein levels of PC were significantly downregulated after reperfusion in hearts subjected to 24 h of hypothermic ischemia. PC-mediated bioprocesses are energy-consuming processes, which may overconsume the energy during reperfusion. But at the same time, the downstream products of PC have an important role in mitochondrial redox homeostasis. We next confirmed the effect of the positive protective role of PC in donor ischemia–reperfusion using oxidative stress assays, serum enzymatic assays, and apoptosis assays in preference to the negative energy-consuming role. Overall, in vivo and in vitro experiments demonstrated that PC deficiency exacerbated oxidative stress as well as apoptosis in the donor heart.

ROS and GSH are the two main substances that maintain cellular redox homeostasis. The excessive generation of reactive oxygen species by the mitochondrial respiratory chain causes an imbalance in redox status. During this process, glutamine metabolism maintains oxidative stress balance by increasing GSH levels [28]. It has been demonstrated that the loss of GSH disrupts cellular redox homeostasis, leading to ROS accumulation, which can result in cell damage and death. Given the fundamental role that glutamine metabolism plays in cell and mitochondrial energy metabolism, glutamine plays a protective effect during IRI. During ischemia–reperfusion, there is a decrease in glutamine metabolism, leading to significant reductions in glutamate levels, glutamine dehydrogenase activity, α-ketoglutarate, and glutaminase mRNA [15]. Our research found that reducing PC levels led to a decrease in GS mRNA expression and glutamine levels, ultimately worsening mitochondrial dysfunction and myocardial IRI.

The Wnt signaling pathway is a highly conserved pathway that controls numerous physiological and pathological processes in the cardiovascular system, encompassing both canonical and non-canonical pathways [29]. The activation of the standard Wnt signaling pathway results in a decline in the stability of β-catenin, causing it to move into the nucleus to bind with TCF/LEF transcription factors and ultimately boost gene transcription [30]. Furthermore, there is increasing evidence supporting the significant involvement of the canonical Wnt/β-catenin pathway in cardiovascular functions, including the control of cardiogenesis, cardiac regeneration, pathological cardiac remodeling, and myocardial IRI [31,32,33,34,35]. We found that the knockdown of PC was accompanied by the inhibition of the Wnt/β-catenin pathway and reduced glutamine metabolism levels. In contrast, the blockade of the Wnt/β-catenin pathway in the presence of PC overexpression inhibited the effective rebound of glutamine metabolism levels. This demonstrates that PC regulates glutamine metabolism through the Wnt/β-catenin pathway.

Recent progress in reducing myocardial IRI in donor hearts exposed to extended cold ischemia time has centered on the mitochondria of donor hearts, with interventions such as medication, gas therapy, and mitochondrial transplantation [20,36,37,38]. Consistent with the literature, we found that PC, the biotin-dependent mitochondrial enzyme, could become a therapeutic target for preventing and treating IRI in heart transplantation. The Wnt/β-catenin pathway serves as a bridge for PC to achieve cardioprotective effects by regulating glutamine metabolism. The results suggest that early overexpression of PC by gene vectors in donor hearts and adding Wnt/β-catenin pathway activators or glutamine to organ preservation fluids have potential therapeutic value in attenuating ischemia–reperfusion injury in heart transplant donor hearts. Nevertheless, to obtain precise targets for interfering with the Wnt/β-catenin pathway, we need to investigate further the specific mechanisms by which PC regulates the Wnt/β-catenin pathway. In addition, therapies that achieve ischemia–reperfusion-protective effects in the donor heart by modulating the PC-Wnt/catenin-GS axis also hold promising prospects for the treatment of myocardial infarction or transplantation of other organs.

## 5. Conclusions

PC expression is downregulated in IRI hearts, while PC treatment ameliorates myocardial IRI in heart transplantation by reducing oxidative stress and cardiomyocyte apoptosis and improving mitochondrial function. The protective effects on the heart seem to be mainly linked to the regulation of glutamine metabolism through the Wnt/β-catenin pathway. Collectively, our findings suggest that PC may become a new therapeutic target for mitigating myocardial IRI in heart transplantation.

## Data Availability

The authors confirm that the data supporting the findings of this study are available within the article.

## Abbreviations

IRI, ischemia–reperfusion injury; PC, pyruvate carboxylase; TCA, tricarboxylic acid; MDA, malondialdehyde; AAV, adeno-associated virus; SOD, superoxide dismutase; BCA, bicinchoninic acid; IL-1β, interleukin-1β; GPX, glutathione peroxidase; IL-6, interleukin-6; LDH, lactate dehydrogenase; MCP-1, monocyte chemoattractant protein-1; ELISA, enzyme-linked immunosorbent assay; NADP, nicotinamide adenine dinucleotide phosphate; TNF-α, tumor necrosis factor-α; NADPH, nicotinamide adenosine dinucleotide phosphate; GSSG, glutathione oxidized form; GSH, L-Glutathione; Bax, Bcl-2-associated X protein; RIPA, radioimmunoprecipitation assay; Bcl-2, B cell lymphoma/lewkmia-2; GS, glutamine synthase; GAPDH, Glyceraldehyde-3-phosphate dehydrogenase; UW, University of Wisconsin; DMEM, Dulbecco’s modified Eagle’s medium; NRCMs, neonatal rat cardiomyocytes; FBS, fetal bovine serum; H/R, hypoxia–reoxygenation; MOI, multiplicity of infection; siRNA, Short interfering RNA; TUNEL, terminal deoxynucleotidyl transferase dUTP nick-end labeling; DHE, dihydroethidium; PCR, polymerase chain reaction; ROS, reactive oxygen species; OXPHOS, oxidative phosphorylation; SD, standard deviation of the mean
